# Supplementary material for: Identification of photoperiod-induced specific miRNAs in the adrenal glands of Sunite sheep (Ovis aries)
Source: Front Vet Sci. 2022 Jul 22;9:888207. doi: 10.3389/fvets.2022.888207 (PMC9354845; doi:10.3389/fvets.2022.888207)
Supplement: Supplementary file 1 [file Data_Sheet_1.ZIP › Supplementary file/miRDeep2 file.zip!/miRDeep2 file/Novel_Structure/SP42A2/Novel_159.pdf]

Provisional ID	:	NC_019463.2_15595
Score total	:	1.6
Score for star read(s)	:	-1.3
Score for read counts	:	0
Score for mfe	:	1.3
Score for randfold	:	1.6
Score for cons. seed	:	
Total read count	:	88
Mature read count	:	88
Loop read count	:	0
Star read count	:	0

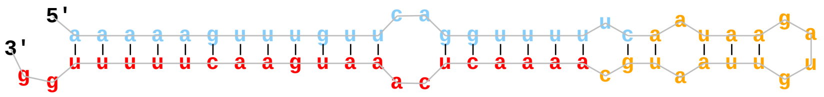

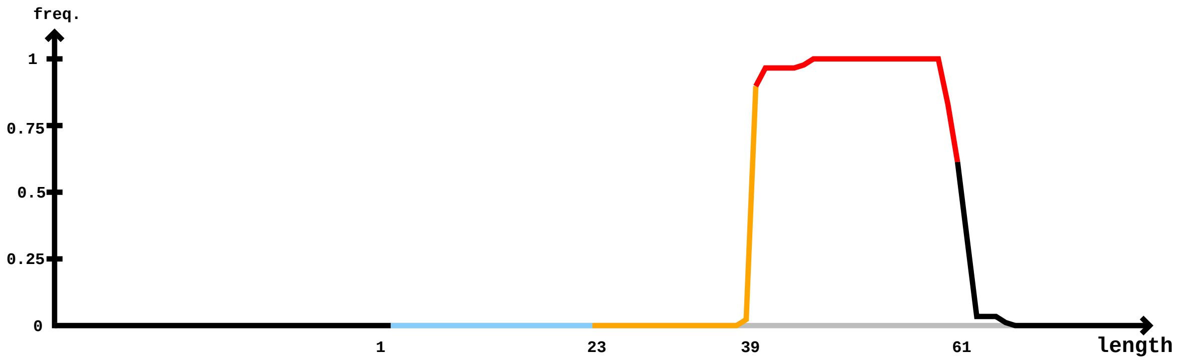

Star Mature

```
5'- aaagcaauuguuuaccuagucauuggguuggcuaaaaaguuuguucagguuuuucaauaagauguuaaugcaaaacucaaaugaacuuuuuggccaacacaaucuuaagcaa   -3'    exp
    .......(((((((...((..((((.(((((((((((((((((((..((((((.((.(((....))).)).))))))..))))))))))))))))))).)))))))))))))    reads    mm    sample
    ......................................................................caaaacucaaaugaacuuuuug....................    1        0     seq
    ......................................................................caaaacCcaaaugaacuuuuug....................    1        1     seq
    .......................................................................aaaacucaGaugaacuuuuu.....................    6        1     seq
    .......................................................................aaaacCcaaaugaacuuuuu.....................    9        1     seq
    .......................................................................aaaacCcaaaugaacuuuuug....................    16       1     seq
    .......................................................................aaaacucaaaugaacuuuuugg...................    2        0     seq
    .......................................................................aaaacucaGaugaacuuuuugg...................    43       1     seq
    .......................................................................aaaacucaaaugaacuuuuuggA..................    1        1     seq
    ........................................................................aaacucaGaugaacuuuuug....................    1        1     seq
    ........................................................................aaacAcaaaugaacuuuuuggc..................    2        1     seq
    ........................................................................aaacucaaaugaacuuuuuggU..................    2        1     seq
    ........................................................................aaacCcaaaugaacuuuuuggc..................    1        1     seq
    ............................................................................ucaaaugaacuuuuuggccaU...............    1        1     seq
    .............................................................................caaaugaacuuuCuggccaa...............    1        1     seq
    .............................................................................Uaaaugaacuuuuuggccaac..............    1        1     seq
```
